# Forest Dynamics and Their Phenological Response to Climate Warming in the Khingan Mountains, Northeastern China

**DOI:** 10.3390/ijerph9113943

**Published:** 2012-10-31

**Authors:** Hongyan Cai, Shuwen Zhang, Xiaohuan Yang

**Affiliations:** 1 State Key Laboratory of Resources and Environmental Information Systems, Institute of Geographic Sciences and Natural Resources Research, Chinese Academy of Sciences, Beijing 100101, China; Email: caihy@igsnrr.ac.cn; 2 Northeast Institute of Geography and Agroecology, Chinese Academy of Sciences, Changchun, Jilin Province 130012, China; Email: zhangshuwen@neigae.ac.cn

**Keywords:** climate change, phenology, deciduous forest, elevation, Khingan Mountains

## Abstract

The Khingan Mountain region, the most important and typical natural foci of tick-borne encephalitis (TBE) in China, is the largest and northernmost forest area and the one more sensitive to climate change. Taking this region as the study area, we investigated the spatio-temporal dynamics of deciduous broadleaf forest (DBF) and its phenology changes in relation to climate change and elevation. Based on MODIS Enhanced Vegetation Index (EVI) time series over the period of 2001 to 2009, the start-of-season (SOS), length-of-season (LOS) and another two vegetation variables (seasonal amplitude (SA) and integrated EVI (SI)) were derived. Over the past decade, the DBF in Khingan Mountains has generally degraded and over 65% of DBF has experienced negative SA and SI trends. Earlier trends in SOS and longer trends in LOS for DBF were observed, and these trends were mainly caused by climate warming. In addition, results from our analysis also indicated that the effects of temperature on DBF phenology were elevation dependent. The magnitude of advancement in SOS and extension in LOS with temperature increase significantly increased along a raising elevation gradient.

## 1. Introduction

The forest ecosystem as an important natural resource provides valuable ecological and socio-economic functions to society, and its changes can influence public health [1,2]. Through the exchange of energy and water vapor between land surfaces and the atmosphere, Gao *et al. *[3] found that only 5 km^2^ of deforestation could cause a significant warming in the climate. Further induced climate changes can alter the risk of public exposure to extreme weather [4,5], climate related diseases and allergenic pollen. Recent studies have also documented the close correlations between the quantity and spatial distribution of pollen with climate [6], as well as of nephropathia epidemica with forest phenology [7]. 

Vegetation phenology examines the life-cycle phases of plants, such as the timing of leaf unfolding, flowering and leaf fall, in relation with environmental parameters. The shifts of plant phenology are closely linked to climate change, especially with temperature in mid-high latitudes [8,9]. Using field observations at the species level, Chen *et al.* [10] reported that a 1 °C increase in late winter and spring temperature has induced 5–6 days’ advance in the the growing season beginning date in China. Avolio *et al.* [11] also found a close relationship between temperature and flowering duration of olive trees. With satellite-based observations, Zhou *et al.* [12] indicated that an extension of the growing season in Eurasia and America was consistent with a warming climate. Although the effects of temperature change on plant phenology have been well documented [13], few studies have explored the sensitivity of phenological responses to temperature gradients related to elevation [14]. For the correlation of disease transmission and elevation [15], phenology changes at different elevation gradients may vary the influence on the public, therefore, studies on spatio-temporal forest dynamics, especially characteristics related to public health, are crucial to assess the implications of any future environmental changes.

The Khingan Mountain region is the largest and northernmost forested area in China, and it is also the most important and typical natural foci of tick-borne encephalitis (TBE) [16], accounting for more than 90% of the whole country’s incidence [17]. TBE is a zoonotic and potentially fatal disease and the causal virus parasitizes common rodent species which preferably inhabit deciduous forests [17]. In addition, the Khingan Mountains are located in the transitional zone between boreal and temperate forests that are more sensitive to climate change. Over the past several decades, this region has experienced a great increase in temperature, and its warming amplitude was much higher than the average level in China [18]. Based on these backgrounds, this study examined the dynamics of deciduous broadleaf forest (DBF) growth, and its phenology response to temperature change during 2001–2009. The objectives of this study were threefold: (1) to investigate spatio-temporal patterns of DBF growth in Khingan Mountains; (2) to quantitatively evaluate the effects of temperature change on DBF phenology; (3) to explore the sensitivity of phenology response to temperature gradients in relation to elevation. This study will improve our understanding of the influences of climate change on montane ecosystems and also help to assess the effects of future environmental changes on public health.

## 2. Materials and Methods

### 2.1. Study Area

**Figure 1:**
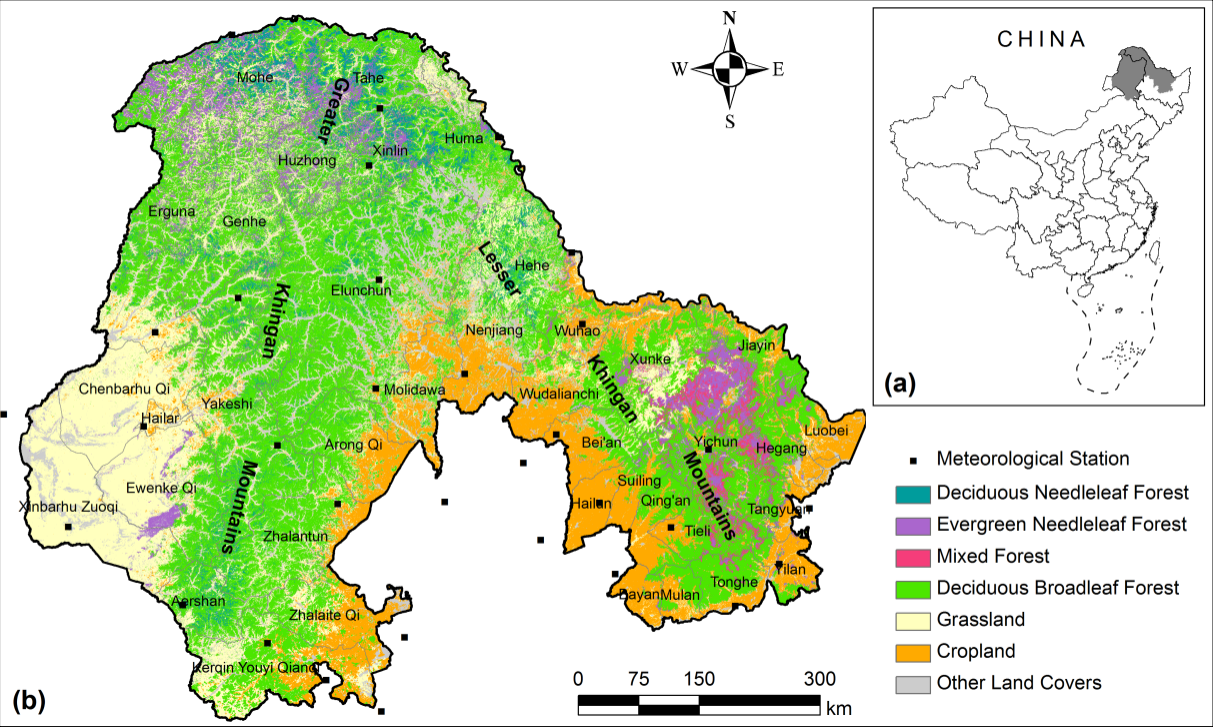
(**a**) The location of the Khingan Mountain region in China. (**b**) Land cover in the Khingan Mountains [19].

The study area is in the Khingan Mountains (117°33′E~131°34′E, 45°41′N~53°33′N), administratively involving Heilongjiang province and Inner Mongolia (approximately 47.3 × 10^4^ km^2^) (Figure 1). The region spans cold and moderate temperate continental monsoon climate zones, with annual average temperatures ranging from −3.7 °C in the north to 4.6 °C in the south. The annual average precipitation increases from 300 mm in the west to 500 mm in the east, while the majority of annual precipitation is concentrated in the summer and autumn growing seasons. 

The Khingan Mountain region includes two parts, the Greater and Lesser Khingan Mountains (GKM and LKM). The GKM, with its ridges running from the northeast to southwest, mainly rises between 1,100 and 1,400 m and is characterized by different topographical conditions on the east and west slopes. The east slope is steep, and adjacent to the productive cropland in the northeast plain of China. The west slope is relative smooth, and adjacent to the Hulunbeier grasslands. The LKM, with its ridges running from the northwest to southeast, rises between 600 and 1,000 m and is characterized by steep southeast slopes and smooth northwest slopes.

The Khingan Mountains host a diverse collection of forest types, ranging from boreal taiga forest to temperate broadleaf forest. Deciduous broadleaf forest (DBF) is the major type, covering over 50% of the forest area [19], and is mainly distributed at elevations between 350 and 900 m. The dominant tree species include Mongolia oak (*Quercus mongolica*), birch (*Betula platyphylla* Suk.), aspen (*Populus davidiana* Dode.) and basswood (*Tilia amurensis* Rupr.). Major coniferous forest species include larch (*Larix gmelini*), Korean pine (*Pinus koraiensis*), fir (*Abies nephrolepis*) and spruces (*P. koraiensis and P. jezoensis*).

### 2.2. MODIS Imagery

The MODIS MOD13Q1 products with a spatial resolution of 250 m, from 2001 to 2009, were collected and produced at 16-day interval using bidirectional reflectance distribution function (BRDF), constrained-view angle-maximum value composite (CV-MVC), and maximum value compositing (MCV) methods [20]. MOD13Q1 includes two vegetation indices (VIs), normalized difference vegetation index (NDVI) and enhanced vegetation index (EVI), and quality data that indicate the quality of the VIs and input reflectance data. Recent comparison analysis of NDVI and EVI showed that EVI had much stronger relationship with towered gross primary production (GPP) and better depiction to the seasonality of GPP [21]. In this study, EVI data were used and reprojected from native Sinusoidal projection to the Albers Conical Equal Area projection.

### 2.3. Meteorological Data

The temperature data were collected from the National Resources and Environmental Scientific Data Center (RESDC) of the Chinese Academy Sciences (CAS). The daily temperature data from 32 weather stations inside and around the Khingan Mountain region over the period 2001–2009 were processed (Figure 1). Average annual temperatures were calculated for each year based on the daily weather records. In order to investigate the relationships between phenology and temperature by pixel, average annual temperature was interpolated into a 250 m by 250 m grid using a Kriging method. 

### 2.4. Phenological Variables

In order to find out the variations of DBF growth and development over the recent decade, two vegetation variables—seasonal amplitude (SA) and integrated EVI (SI)—were extracted from the MODIS-EVI time series data. SA was calculated as the difference between the maximum and base EVI value over the season. SI was defined as area under the vegetation curve and above the base level during the growing season. Several studies have suggested SA and SI are closely related to Net Primary Production (NPP) during the growing season [22]. An analysis of grasslands in the United States indicated a positive and statistically significant relationship between integrated NDVI and field aboveground NPP [23]. Thus, SA and SI provide good indicators for capturing the activity of vegetation growth over the season. Other two phenological variables were also estimated: the start-of-season (SOS) were estimated with the threshold of 20% SA [24] and length-of-season (LOS) corresponded to the date difference between SOS and end-of-season. 

The Timesat (v. 3.02) software was used to smooth EVI time series and extract vegetation and phenological variables with a double logistic function [25,26]. The program was designed to derive phenological information from time series of satellite data by fitting their upper envelopes with multiple processing methods. The MOD13Q1 pixel reliability band was used to weight each value at same pixels in the time series ensuring that clouds and other low quality data had minimum effects to the fittings. The weight of good data was set to 1.0, marginal data to 0.5 and poor quality data to 0.1.

The DBF pixels with no significant seasons due to data noises failed to extract vegetation and phenological variables, and were removed from the analysis. Additionally, the resultant grids with abnormal phenology values (e.g., SOS ≤ 0 or LOS > 360) were also removed from the trends analysis. 

### 2.5. Statistical Analysis

In order to determine spatial patterns of directions and rates of change, trends in phenological variables through time and temperature were computed by an ordinary least squares regression method, and the trend slopes were estimated and mapped by pixel. For measuring the significance of the estimated trends, all trends exceeding the respective sigma error were labeled as significant trends. In addition, trends in SA and SI were expressed in percentage relative to the value of the linear trendline at starting point of the time series. 

## 3. Results

### 3.1. The Vegetation and Phenology Change of DBF

**Figure 2:**
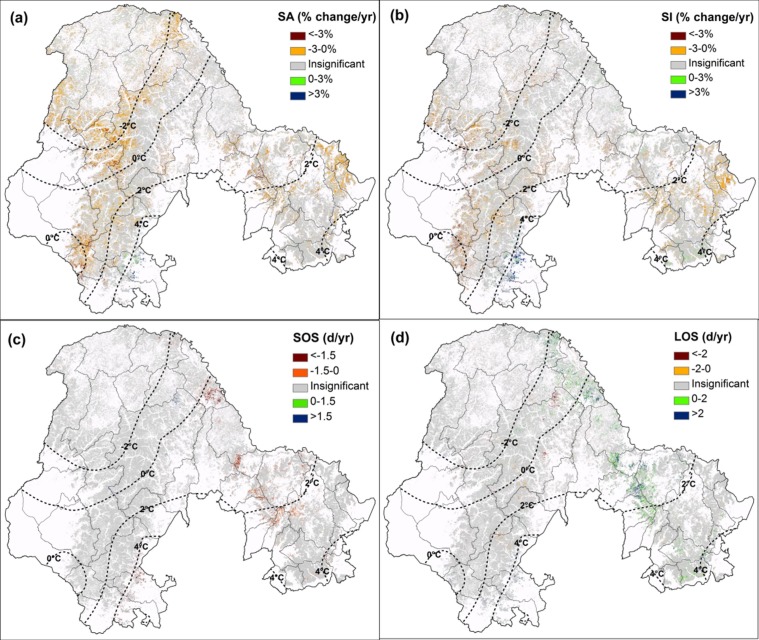
Significant trends in (**a**) SA, (**b**) SI, (**c**) SOS, (**d**) LOS for DBF in the Khingan Mountains. The trends described here were statistically significant at the 95% confidence level.

The spatial patterns of trends in vegetation and phenological variables for DBF in the Khingan Mountains are shown in Figure 2. 

For SOS, the positive and negative trend indicated later and earlier dates, respectively. From 2001 to 2009, SA and SI for DBF in the Khingan Mountains have generally decreased, which indicates the loss of net primary DBF production. SA has decreased over about 75% of DBF, among which 13.38% is statistically significant (*p * < 0.05) (Table 1). The decreasing trends are extensively distributed, however, the strongest increasing trends (more than 3% per year) are mainly focused in the southeast of GKM, in the counties of Zhalantun, Zhalanteqi and Kerqin Youyi Qianqi. Trends in SI had a similar pattern to that of SA, but less extensive in the decreasing regions and much stronger and extensive in the increasing regions, which is probably because SI correlates with SA, as well as the season length [27,28]. 

In general, earlier SOS and longer LOS trends have been detected in the DBF. The green-up onset dates have advanced in more than 70% of the DBF, although only 1.93% is statistically significant (Table 1). Patches with earlier trends in SOS were mainly located in the LKM and the southern GKM where the TBE incidence was much higher [29]. The largest patches were located in Huma County. Trends of LOS were dominated by variations in SOS, and the spatial patterns of trends in LOS were similar to those of SOS, but with contrary change direction, that is, the earlier SOS corresponding to longer LOS and *vice versa*. The longer trends were mainly located in the LKM and Tahe-Huma-Heihe counties and the shorter trends were scattered around GKM. 

**Table 1:** The proportions of pixels with statistical trends to total DBF for four variables.

Variables	Negative trends		Positive trends		Total	
SA	13.38 ^a^	75.32	0.93 ^a^	24.68	14.31^ a^	100
SI	7.99 ^a^	66.39	1.86 ^a^	33.61	9.85 ^a^	100
SOS	1.93 ^a^	70.81	0.3 ^a^	29.19	2.23 ^a^	100
LOS	1.16 ^a^	39.99	3.74 ^a^	60.01	4.90 ^a^	100

^a ^Statistically significant trends at the 95% confidence level.

### 3.2. The Impact of Temperature on Phenology Change

#### 3.2.1. The Temperature Change in Khingan Mountains

To reduce the effects of landscape heterogeneity on trend analysis of temperature, all 32 climate stations were grouped into four classes according to the elevation and latitude (Table 2). 

**Table 2:** Temporal trends of average annual temperature among climate stations.

Classes	Slope	R^2^	*p*	Number of stations	Elevation (m)	Latitude
Class1	0.245	0.55	0.022 ^a^	15	0–400	45°N–49°N
Class2	0.228	0.56	0.021 ^a^	8	0–400	49°N–54°N
Class3	0.215	0.53	0.025 ^a^	4	400–1,200	45°N–49°N
Class4	0.209	0.51	0.032 ^a^	5	400–1,200	49°N–54°N
Total stations	0.227	0.53	0.027 ^a^	32	0–1,200	45°N–54°N

^a ^Statistically significant trends at the 95% confidence level.

**Figure 3:**
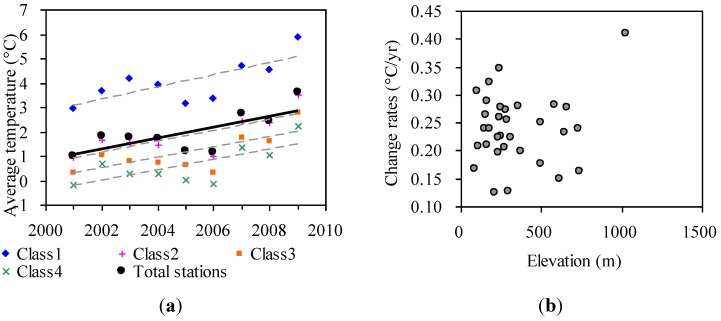
(**a**) Trends of average annual temperature during 2001–2009, (**b**) and the relations with elevation over 32 climate stations. The regression lines of temperature on year have been superimposed in (a).

During the period from 2001 to 2009, there was a significant increase in average annual temperature (R^2 ^= 0.53, *p *= 0.027) in the entire region, with a linear trend of approximately 0.23 °C per year (Figure 3(a) and Table 2). The temperature increases have been widespread in the region, and were observed in all 32 climate stations, with the warming rates ranging mainly between 0.1 °C and 0.45 °C per year, but no significant correlations were observed between the warming rates and elevation (Figure 3(b)).

#### 3.2.2. Statistical Patterns of Relationships between Phenology and Temperature

To demonstrate the relationship between temperature and DBF phenology, trend slopes were estimated by pixel. A negative relationship indicates that as temperature increases, SOS occurs earlier and LOS becomes shorter, respectively. About 56.86% of the DBF had a negative relationship between SOS and temperature, that is, as the temperature increased during 2001 to 2009, the advance trends in SOS (more than 70%) being mainly explained by the warming climate. The positive relationship between temperature and LOS was more apparent, covering 65% of the DBF. 

#### 3.2.3. The Effects of Temperature Gradients in Relation to Elevation

**Figure 4:**
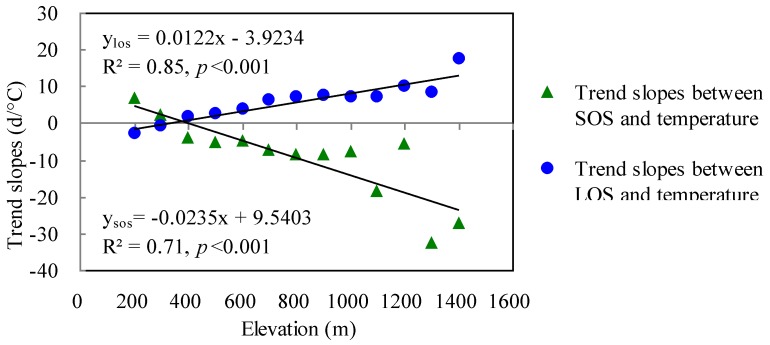
The trend slopes of phenological variables with temperature as a function of elevation.

Across the elevation range from 200 to 1,400 m in the Khingan Mountains, the relationships between temperature and SOS were negative and they were positive with LOS above 400 m. The trends have increased with the increase in elevation (Figure 4), that is, as the elevation increases, the phenological responses of DBF to temperature become more sensitive. The relations of elevation to the relationships between temperature and SOS or LOS were all statistically significant at the 95% confidence level. Based on the assumption that temperature decreases when elevation increases, every 100 m increase in elevation has induced the earlier rates of SOS following temperature decrease to increase 2.35 d/°C (R^2 ^= 0.59, *p *< 0.001), and longer rates of LOS to increase 1.22 d/°C (R^2 ^= 0.87, *p *< 0.001) (Figure 4).

## 4. Discussion and Conclusions

Taking the Khingan Mountains as the study area, this study explicitly investigated the spatial dynamics of DBF and its phenology in relation to climate warming and elevation. Our efforts are not only devoted to improve our understanding of the influence of climate change on montane ecosystems, but also to provide information on spatio-temporal patterns of the main habitat of the TBE host and this information will support risk analysis and prevention of TBE. 

Using SA and SI for characterizing seasonal development of DBF in Khingan Mountains, we found decreasing trends with spatial variation over the past decade, and this result confirms previous findings in the northeastern China region by Guo *et al.* [30]. This decreasing trend may be caused by several factors. Firstly, the drought stress due to sudden increases in temperature (Figure 3(a)) and decreased precipitation in the Khingan Mountains [31] could complicate the impacts of a warming climate on vegetation activity. Furthermore, a warming climate also increases forest wildfire risk [32] and closely relates to the areas of insect pest occurrence [33], which could also contribute to the decreasing trend seen in the Khingan Mountains. 

This study observed a trend of extension of the growing season in the Khingan Mountains, and this trend was mainly explained by temperature increases during the recent decade. This finding was in accordance with the results from *in situ* observations [34], remote sensing data [35] and phenological models [36]. According to Chen *et al.* [37], the growing season in the northern temperate China regions (with latitudes higher than 44°N) has been extended significantly from 1982 to 1993, and this extension was caused by an advance in SOS and delay in the end of growing season. In this study, a further extension of growing season was detected from 2001 to 2009 in the Khingan Mountains. Moreover, this extension trend was mainly dominated by the variation of SOS (Figure 2). 

Our analysis also revealed that the effects of temperature on forest phenology depended on elevation. During the recent decade, annual mean temperature has significantly increased over the Khingan Mountains and the warming rates have no significant relations to elevation. However, the impacts of temperature on DBF phenology were enhanced with increase in elevation. The effect of elevation on phenology change has been documented in previous studies [9]. For instance, Vitasse *et al. *[14] indicated that leaf phenology in European trees was delayed 1.1–3.4 days for every 100 m increase in elevation. This study further found that the advance responses in SOS and extension responses in LOS with temperature increase were more sensitive at higher elevations. This indicated that a 1°C increase in temperature at a higher elevation would cause more of an advance in SOS and extension in LOS than at a lower elevation. With the projections of climate warming in the future, the re-adjustment of the effects of elevation on phenology patterns induced by temperature change will vary over elevation gradients. 

Although TBE is an acute, potentially fatal infectious disease and a great threat to public health, studies on the distribution of the TBE risk area and the transmission mechanism associated to environmental change were lacking in China [17]. Previous studies have indicated that changes of vegetation phenology influence rodent populations [38], and Barrios *et al.* [7] further found a connection between nephropathia epidemica occurrence and specific trends in the phenology of broadleaf forests in Belgium. Based on satellite-derived phenology variables and spatial statistical analysis method, this study provided spatially and temporally explicit patterns on phenology dynamics of DBF and its response to warming climate. This information will be useful for identifying areas at risk of TBE and preventing TBE outbreaks.
